# Robot-Mediated Nudges for Workplace Health: Not a One-Size-Fits-All Modeling Problem

**DOI:** 10.1007/s12369-023-01086-x

**Published:** 2023-12-26

**Authors:** Rhian C. Preston, Kenna Dinsdale, Madison R. Shippy, Naomi T. Fitter

**Affiliations:** 1 CoRIS Institute, Oregon State University, P.O. Box 1212, Corvallis 97331, OR, USA

**Keywords:** Socially assistive robots, Robot nudges, Personalization, Markov Decision Process models

## Abstract

Prolonged sedentary behavior in the vast population of office and remote workers leads to increased cardiovascular and musculoskeletal health challenges, and existing solutions for encouraging breaks are either costly health coaches or notification systems that are easily ignored. A socially assistive robot (SAR) for promoting healthy workplace practices could provide the physical presence of a health coach along with the scalability of a notification system. To investigate the impact of such a system, we implemented a SAR as an alternative break-taking support solution and examined its impact on individual users’ break-taking habits over relatively long-term deployments. We conducted an initial two-month-long study (*N* = 7) to begin to understand the robot’s influence beyond the point of novelty, and we followed up with a week-long data collection (*N* = 14) to augment the dataset size. The resulting data was used to inform a robot behavior model and formulate possible methods of personalizing robot behaviors. We found that uninterrupted sitting time tended to decrease with our SAR intervention. During model formulation, we found participant responsiveness to the break-taking prompts could be classified into three archetypes and that archetype-specific adjustments to the general model led to improved system success. These results indicate that break-taking prompts are not a one-size-fits-all problem, and that even a small dataset can support model personalization for improving the success of assistive robotic systems.

## 1 Introduction

Work-from-home arrangements have become increasingly common over time, and the COVID-19 pandemic drastically increased the rates of remote work to heights that have not (and may never) return to pre-pandemic levels. In both remote work and general sedentary office work situations, health challenges such as poor cardiovascular and musculoskeletal health are a common issue for computer users, who often fail to take sufficient breaks from sitting [1]. Corporations, in turn, have explored solutions such as phone apps [2] and computer notifications [3] to address this sedentary behavior. Research on these approaches to date show that screen-based solutions quickly fall into disuse and lack a peer-like social component [2], which is generally difficult to scale up. In past work, our research group proposed socially assistive robots (SARs) as an alternative break-taking support solution with the potential to offer the scalability of apps along with the heightened motivation of a health coach [4]. The present follow-up article focuses on model formulation for such a break-taking SAR system, to build on promising preliminary results.

Physically present, or embodied, systems like SARs are well suited for promoting healthy practices; people are more likely to oblige the requests of embodied robotic systems compared to their virtual counterparts [5, 6]. Further, people are more attuned to the motions and changes of embodied systems in their space compared to onscreen agents [7], which implies that users are also more aware of these systems. Existing work on embodied break-taking systems in particular found that participants respond positively to this type of robotic application and are more responsive to systems that are perceived as more social, but that users also have distinct opinions about how these systems should behave within their workspace [4, 8]. Relatedly, a past long-term examination of user break-taking habits revealed both individual and generalized trends among participants [9], which hints at the need for more sophisticated interaction modeling within this domain. Compared to past related work, the efforts presented in this paper involve a longer-term system deployment and a more nuanced robot behavior model.

**Fig. 1:**
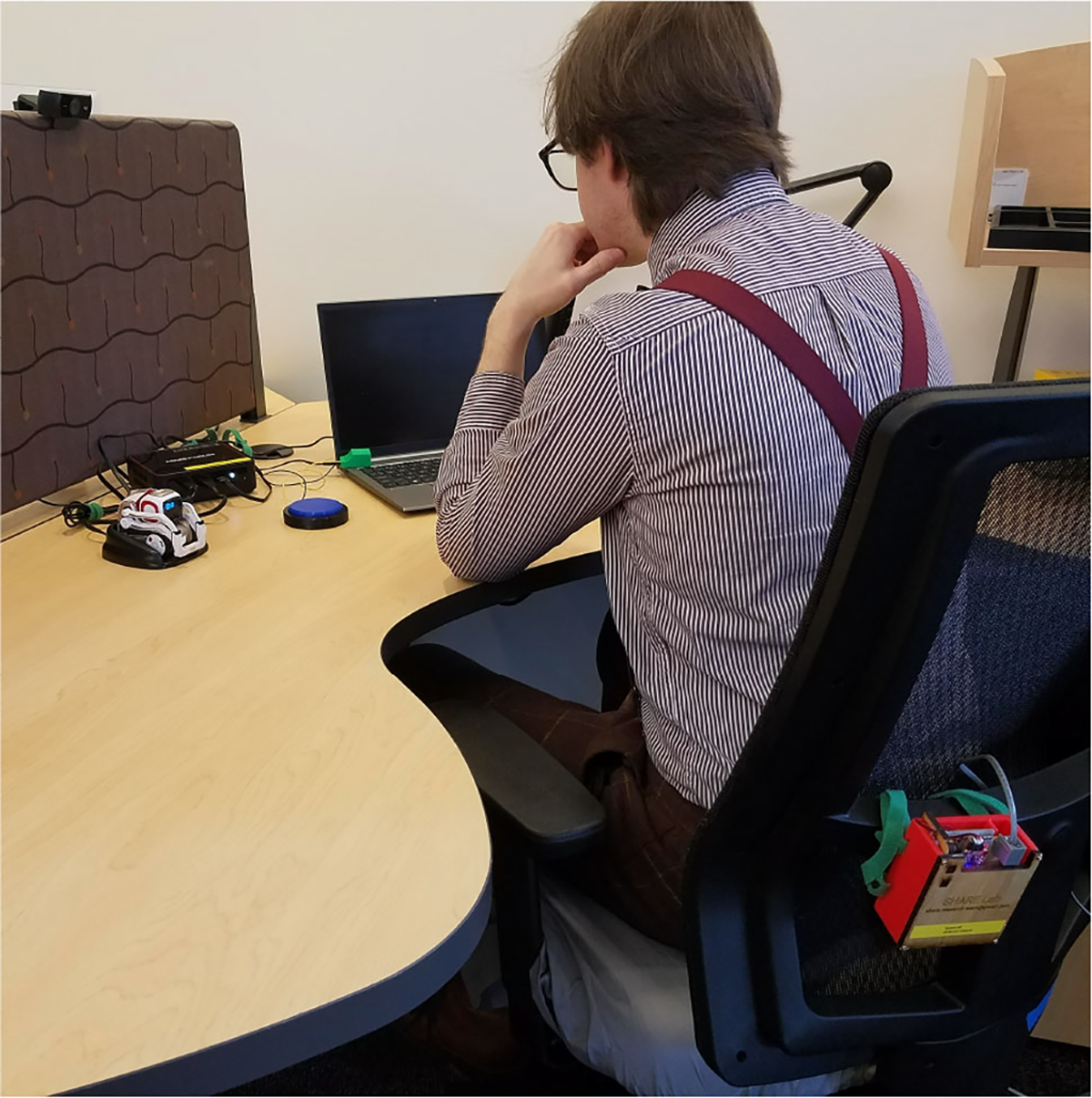
A mock user sitting at their workspace, which is equipped with the break-taking SAR system presented in this article

Prior to the current work, the research team conducted a two-day-long study comparing participant experiences with a robotic standing break buddy vs. a non-embodied break-taking prompt system. Results showed that interaction with the robot option was most pleasant, enjoyable, and engaging, but that the fixed prompt timing and behavior strategy of the robot was one key flaw of the system. Accordingly, our key research goal in the follow-up presented work was to *learn how to formulate behavior models for break-taking robotic systems that promote system success and continuous use.* After reviewing related past literature in Sect. 2, the presented work centers on a long-term data collection, a short-term data collection, and the design of system behavior models based on the collected data. The long-term data collection involved the robotic system presented in Fig. 1 and described in Sect. 3 as part of a two-month-long study of participant responses to different SAR break-prompting behaviors, as further detailed in Sect. 4. The short-term data collection described in Sect. 5 leveraged week-long SAR deployments to gather additional user response data for model development. With the gathered data, we developed the Markov decision process (MDP) model described in Sect. 6, after which we discuss the resulting policies and their effectiveness for individual participants. Section 7 offers a discussion of the key results and concluding thoughts about how to apply these ideas in related robotics efforts. Overall, key contributions of the work include evidence that the proposed intervention tends to successfully reduce sitting time, in addition to a set of participant archetype-based interaction models that can help this type of system personalize successfully and work even more effectively in the future.

## 2 Related Work

We were guided by existing work related to health impacts and mitigation of sedentary behavior, strategies for successful interruptions, and SAR systems for encouraging user behavior changes. The following subsections further explain this important prior work.

### 2.1 Taking Breaks for Health

Both office and remote work employees are predominantly sedentary workers. The wellbeing of these groups has drawn attention in recent years due to the negative health impacts associated with prolonged sedentary periods [10, 11]. There is an abundance of research showing the negative impacts of prolonged sitting, with consequences including (but are not limited to) increases in premature mortality rates [1] and worsened pregnancy outcomes [12]. Expanded research into these negative health impacts explored how they differed depending on the duration of sedentary time (i.e., longer periods of sitting vs. shorter periods of sitting), finding that mortality increases were significantly lower for sitting periods less than thirty minutes [13]. In turn, attention on how to break up sedentary behavior periods has increased. Efforts in this space show that breaks as short as five minutes improve physical and cognitive function for sedentary workers [14]. These works informed the intervention behind our project, in addition to key sitting durations and break periods selected in this effort, as further discussed in later sections.

Based on the negative health effects of sitting and beginning findings on how to improve health outcomes of sedentary workers, subsequent efforts have explored how to encourage breaks. Solutions include approaches using digital health management apps or tracking systems [2, 15–18], as well as training for correct usage of sit-stand desks [11, 19]. These sit-stand desks in particular—when combined in multi-pronged approaches that include training, peer encouragement, and habit tracking—have yielded significant successes in improving job performance, engagement, and quality of life [20, 21]. At the same time, screen-based health tools fall into disuse quickly, and human-mediated solutions are difficult to deliver at scale. Accordingly, our work considers an intervention similar to those of existing digital tools, but with a robot-mediated physical presence in the user’s workspace.

### 2.2 Shaping Beneficial Interruptions

A key tactic to encourage specific human actions or choices is the act of nudging. Nudging, as originally defined in behavioral economics, is any designed aspect of a choice “… that alters people’s behavior in a predictable way without forbidding any options or significantly changing their economic incentives” [22]. Nudging has since been popularized within health care in particular. For example, nudges have been adopted as a new method to encourage individuals to practice healthy behaviors, and are often used in conjunction with other health-promoting methods in larger frameworks [23, 24]. These nudge-based behaviors have been used primarily to direct users toward voluntarily making healthier choices, such as exercising [25] or limiting use of smartphones [26]. We rely on similar principles to encourage break-taking through our investigated robot interactions.

To encourage break-taking, systems like our robot will typically need to navigate prompting people during their day-to-day work, much like any coworker looking to ask a question or start a conversation. Thus, we need to understand both how and when to interrupt an individual in the workplace. How to interrupt a person can be broken into two main modalities: verbal and non-verbal interruptions. Work examining interrupting conversations between multiple people found that participants preferred non-verbal cues before verbal cues [27]. Within the open office spaces used by many modern desk workers, prompting people with non-verbal behaviors also limits disruption to others in the shared space. Non-verbal cues can have differing levels of immediacy and subtleness. These spectra are reflected in the use of always visible systems vs. pop-ups on screens [28], as well as in subtle versus sudden pose changes in embodied systems [7]. It has also been shown that human interlocutors can correctly identify the urgency of non-verbal robot cues patterned off of human interruptions [29]. These non-verbal cues focused on aspects of the interruption such as speed of motion, gaze, head movement, rotation, and proximity to the person. While this and other existing work has focused on perceived urgency of interruptions, these works did not explore the variation in disruption tolerance due to specific tasks or individual user characteristics.

When to nudge individuals is the second challenge. While studies have shown productivity benefits of frequent short breaks [30], other work has demonstrated that these periodic interruptions can lead to more fragmented work [31]. This variation is, at least in part, dependent on the need to focus on a task [32]. Thus, watching for focused attention helps to determine when users are more open to disruption. This strategy was used in short-term learning methods with a past interrupting robotic system [33]. Further work has examined both when to interrupt individuals and when they are most likely to stop or change a task on their own [9]; the research team tracked software activity, mouse movement, and keyboard activity to build a predictive model of when to prompt users via a computer-based interruption system. The related past work supported our beginning understanding of interruption timing strategies; for example, we know that there is a need to time interruptions to avoid periods of high focus [34]. At the same time, there is a notable gap in understanding individual user responses, especially when nudges come from varying robot behaviors. Our work begins to address these open questions.

### 2.3 SAR Systems and Behaviors

SARs for behavior-change is an active area of research which helps to inform the appropriate design of robotic systems that influence user behavior via social interaction [35], such as our proposed break-taking buddy. SAR systems span a broad range of goals and contexts, including encouraging social skill practice [36], physical therapy exercises [37, 38], and daily living tasks [39, 40]. We specifically utilize the break-taking SAR system described in [4] as a basis for the current work, building on the same Cozmo robot as the social agent in the system.

SAR systems’ expressive or emotional behaviors can significantly impact user behaviors and behavior change success [41]. In selecting prompting behaviors for our SAR system, we leveraged past Cozmo robot behaviors that were designed and validated in [42]. These behaviors span the valence and energy axes of Russel’s circumplex model [43], which provided us with variability in the robot nudging behaviors. Based on past participatory design work for break-taking robots, we also noted that participants preferred a system with minimal noise production and a small footprint [8]. These insights helped us to select the non-verbal expression modalities, as discussed later in the article.

## 3 SAR System Design

The robotic system for the data collections was based on the past SAR system from [4]. This past system used the commercial Anki Cozmo robot as the embodied agent for supplying break-taking prompts to the participant and a seat occupancy sensor to detect periods of sedentary behavior. Cozmo served as an ideal robot due to its small form factor, low cost, and considerable expressive ability. From past related work, we additionally had a set of validated expressive behaviors for the Cozmo system [42] which satisfied known requirements for workplace technology, such as muting the robot’s inbuilt audio cues for minimal sound emission [8, 27]. Another advantage of using this system as our basis was the limited invasiveness of the system’s sensor data. By avoiding using a camera in the system and by tracking sedentary behavior without a connection to a user’s personal computer, we could offer more privacy to these individuals, as required in many remote work scenarios. Additional components of the past SAR system were a Raspberry Pi 3 B+ processor and Android phone, which together controlled the robot and occupancy sensor. As further described in the past work, the robot connection for this setup, which relied on the Cozmo SDK, led to occasional disconnects of the robot, which needed to be manually addressed. Lastly, the system included a USB webcam solely for study data collection purposes. In response to the initial system’s programmed behavior, which delivered a break-taking prompt after 30 min of sitting behavior, past study participants shared ideas for improvement of the system’s fit with workflow needs.

For the efforts presented in this article, the SAR system would be operating for much longer periods than in the previous work, and we also sought to address flaws identified by study participants in the past system. Thus, as further described in the following subsections, we needed to change both the controlling hardware and software to be robust enough to operate the robot without direct research team supervision for a two-month period. Additionally, based on the results from [4], we knew that interruptions from the system needed to be timed better to fit in with participant workflow, in addition to including more options for snoozing or slightly postponing a robot nudge. Our related efforts to add further informative (and yet noninvasive) sensing, as well as a snooze button, to the system are detailed below.

### 3.1 Updated System

**Fig. 2:**
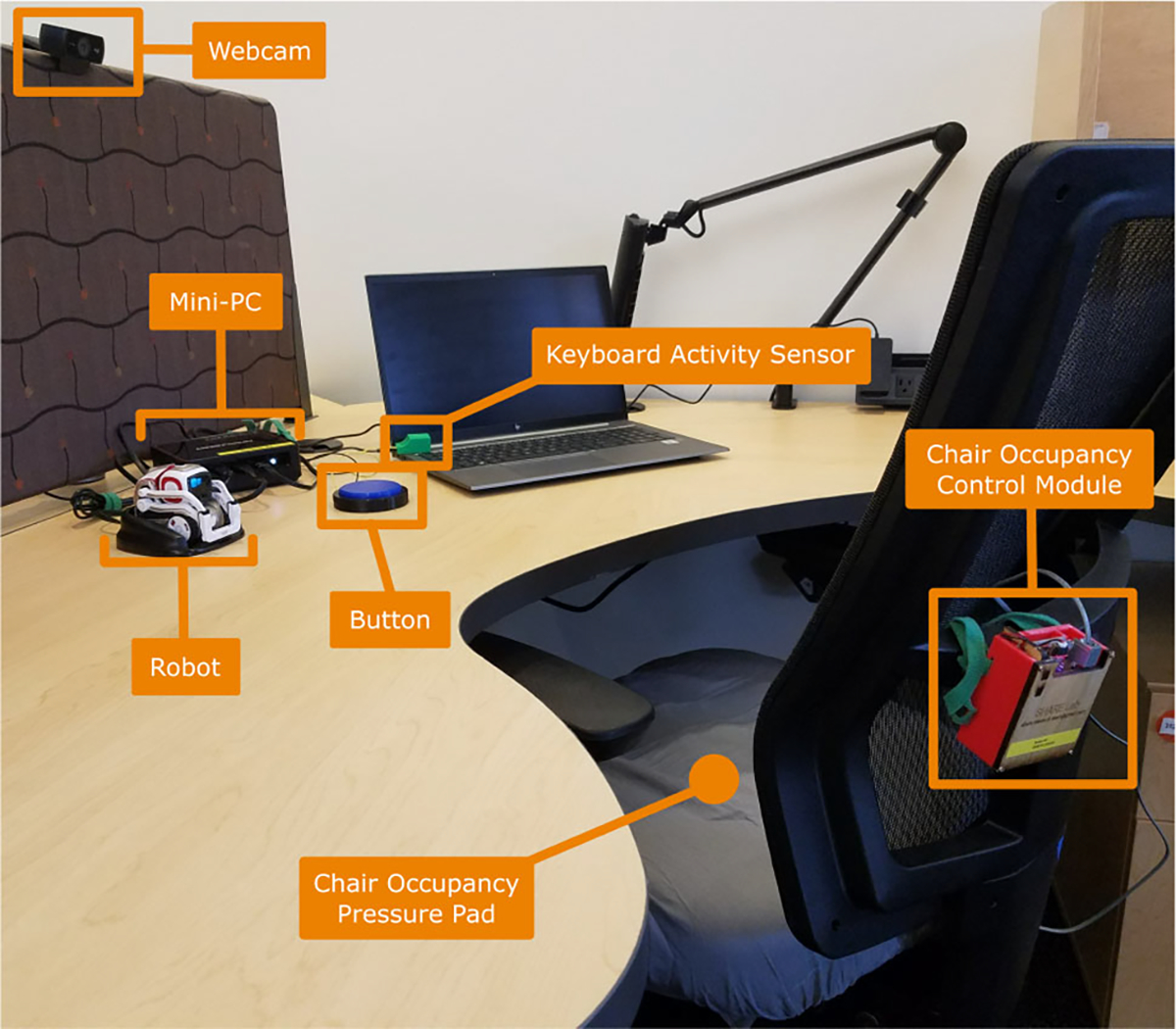
Labeled system components of the SAR break-taking system at a mock user’s desk

The updated SAR system for the present work, as shown in Fig. 2, included the same Cozmo robot and seat occupancy sensor as in the past work, in addition to a newly added mini-PC, keyboard activity sensor, and snooze button.

As in the past work, the small form factor and expressive behaviors offered by the Cozmo robot fit well with the workplace use context. While the commercial Cozmo robot has built-in expressive audio cues, like in the past work, we chose to mute them to satisfy known workplace norms. The past processor and robot control setup, however, required significant updates to be able to support a reliable long-term robot connection. In particular, the official Cozmo SDK, as used in the past system, requires a tethered smartphone; the Android phone used for this purpose in the past setup proved to be unreliable for both staying connected and reconnecting with the robot. Thus, the central robot control hardware was upgraded to the aforementioned mini-PC, which has an Intel i5 processor. The mini-PC runs Ubuntu 20.04 with ROS Melodic and uses the PyCozmo open-source library to interface with the Cozmo robot [44]. This connection method allowed for greatly increased connection stability with the robot, in addition to programmatic reconnection to Cozmo in the case of any disconnects that do occur, which present as brief periods during which the Cozmo face screen is blank.

Based on user critiques from the past investigation and best practices from related work, we changed the previously static interruption timing to instead be a variable timing (within a set 15-minute window) meant to interrupt participants when they were less attentively focused on work. To accomplish the variable interruption timing, we used a simple accelerometer-based keyboard activity sensor as a proxy for focused attention. In addition to the more nuanced interruption strategy, we introduced a button element that allowed the user to “snooze” robot prompts. We used the chair occupancy sensor, a large contact pad that rests under a seat cover and communicates its state (weighted or unweighted) with the mini-PC via Bluetooth, to track length of sitting time. The keyboard activity sensor, a LIS3DH accelerometer connected to the base station via a Teensy 3.2 microcontroller, was affixed to the participant’s keyboard to sense keystrokes as a means for improving the system’s gauge of user interruptibility. Specifically, we used the tap detection mode of the accelerometer to sense time elapsed since the last keystroke, and we provided a prompt after two consecutive minutes of no typing. The halt, or snooze, button is a momentary pushbutton connected to the same Teensy 3.2 microcontroller. Participants could press the button to immediately halt the current Cozmo prompt action.

As in the past work, for study purposes, we used a Logitech 920 1080p webcam to record audiovisual information about how participants responded to the system. This webcam recorded five minutes of audiovisual data upon each delivery of a break-taking prompt.

### 3.2 Updated Operation States and Behaviors

**Fig. 3:**
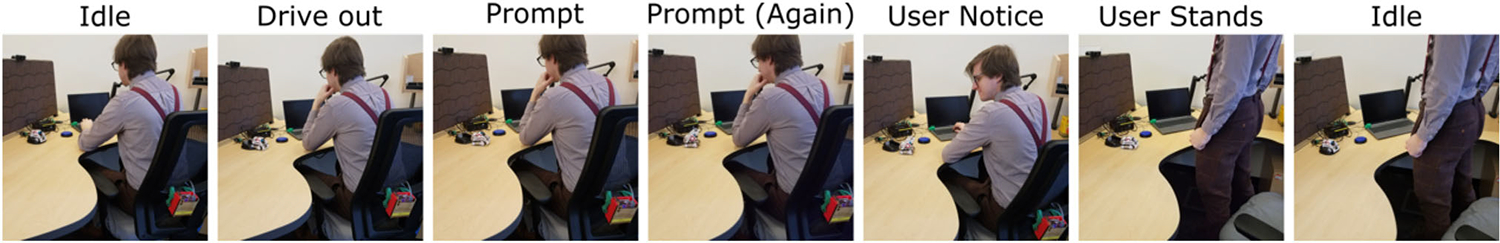
System operation cycle, beginning with the idle state. After approximately 30min of user sitting time, the robot drives out to prompt the user. These prompts repeat at five-minute intervals until the user stands up, at which point the system returns to idle state

As mentioned above, the original Cozmo system supplied a break-taking prompt every 30min, using randomly selected Cozmo behaviors from the set of available actions. The updated break-taking system has a richer set of operation states defined by participant actions, as well as a more clearly defined delineation of robot behaviors. An example operation cycle of the system appears in Fig. 3.

The high-level system operation can be broken down into three key operation states, between which the system transitions depending on user behavior:

- *Idling:* system state when the user is either standing or has sat down but not yet been prompted
- *Prompting:* state during which the system acts to encourage the participant to take a break
- *Snoozing:* state during the five-minute period after a prompt has occurred and been ignored by the user or intentionally halted with the button. (After this delay, the the system will prompt the participant again, returning to the prompting state above.)

**Fig. 4:**
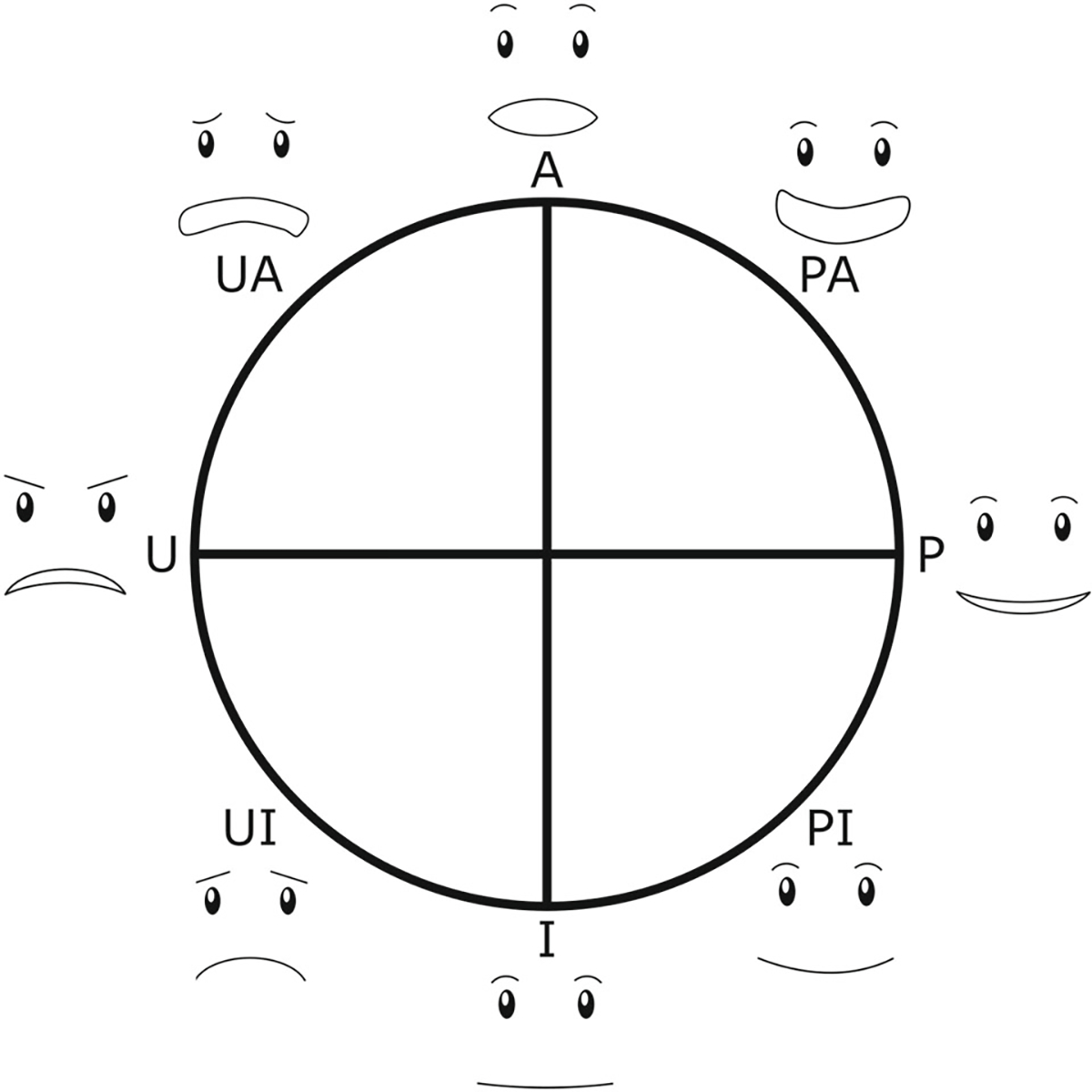
Circumplex model of robot behaviors. The actions are named using combinations of [P]leasant or [U]npleasant and [A]ctive or [I]nactive

The robot itself had behaviors associated with system operation state. During both the idle and ‘snooze’ states, the robot remained still, cycling the ‘blink’ animation of the default eyes on the Cozmo’s LCD screen. During the prompting state, the robot used expressive behaviors to encourage the participant to take a break. These behaviors, from [42], were designed to span varying energy (active or inactive) and valence (pleasant or unpleasant) levels based on Russel’s circumplex model of affect [43]. The alignment of these behaviors with their intended affect was also validated in [42] through an online video-based study. The behaviors belong to eight behavior categories: Active (A), Pleasant Active (PA), Pleasant (P), Pleasant Inactive (PI), Inactive (I), Unpleasant Inactive (UI), Unpleasant (U), and Unpleasant Active (UA), as shown along the circumplex model in Fig. 4. Unpleasant behaviors are often excluded from SAR interactions, but past work has shown that sometimes more unpleasant or impolite behaviors can encourage people to carry out actions that they might be resistant to performing otherwise [45]. Sample behaviors for selected behavior categories appear in the repository associated with this work [46].

**Fig. 5:**
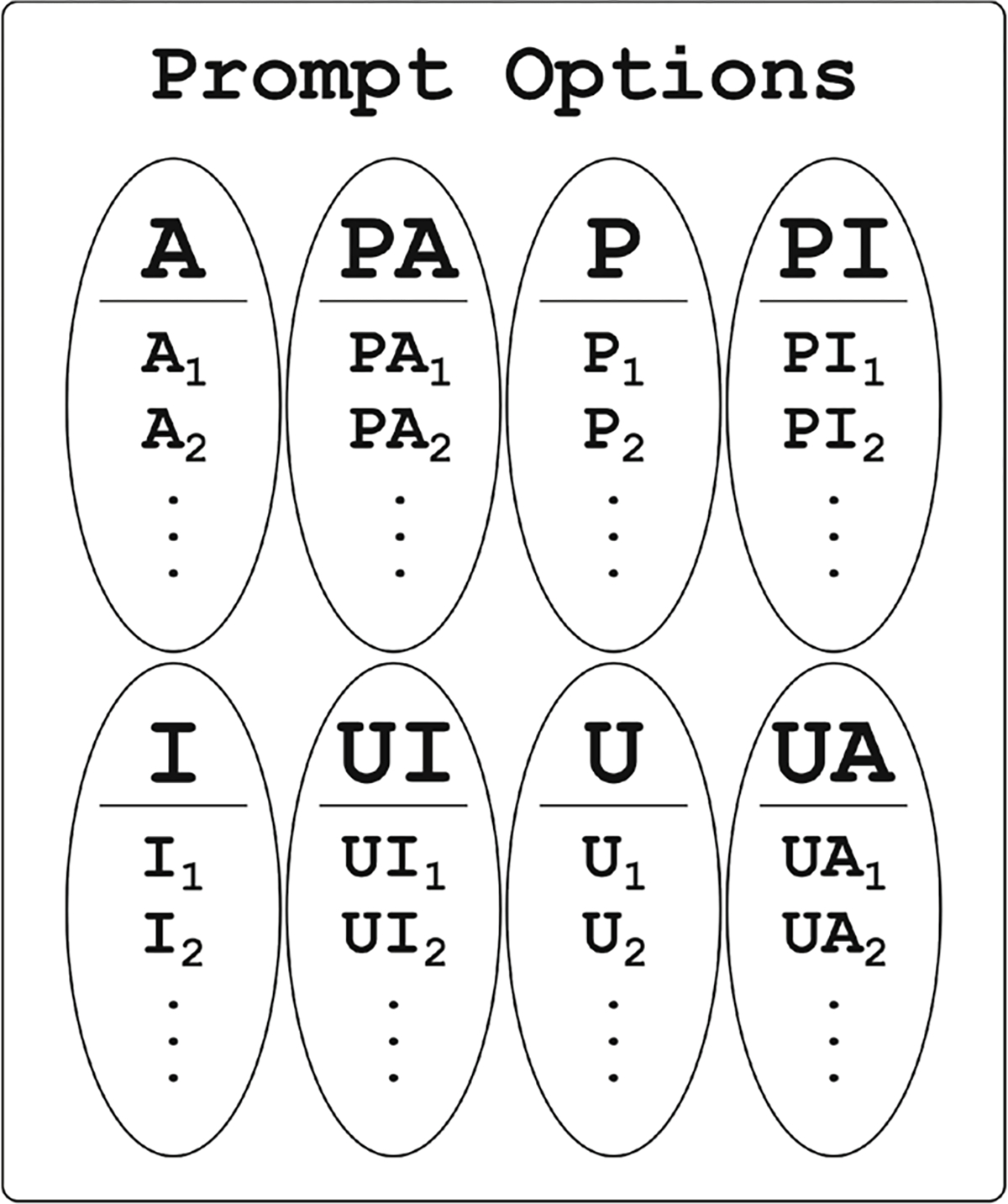
A visual representation of the behavior categories and individual behaviors associated with each category that our robotic system could perform. When prompting the participant, the system randomly selects one behavior category (depicted here as ovals), and then performs three behaviors from within that selected category (depicted here as letters with numbered footnotes)

During each prompting state, one category was randomly selected, and the robot performed three behaviors randomly selected (without replacement) from that specific category, as further illustrated in Fig. 5. This randomization method was chosen to satisfy requirements associated with maintaining a consistent connection to the Cozmo robot in real-world environments, while still traversing the full robot action space. If the participant pressed the halt button or failed to stand up during the robot prompting behaviors, then the robot would return to the ‘blink’ animation, and the system would enter the ‘snooze’ operating state.

## 4 Exploratory Long-Term Deployment

In order to understand how robot nudges can support user break-taking over extended periods of time, we needed to evaluate responses to the updated SAR system in natural day-to-day environments for a duration extending beyond the novelty effect (i.e., a month or longer for adoption [47]). Our previous work with a two-day-long deployment of the system resulted in positive perceptions of the SAR over a non-embodied alternative [4], but it was not clear if this effect would persist over time. The longer-term, in-the-wild study presented in this work allowed for a better understanding of user experience with continuous use over time by deploying the robotic system for a month-long intervention period, in addition to gathering data about each participant’s usual behaviors without the robot nudges. We also gained a vastly increased number of interaction observations per user, which contributed to the modeling efforts discussed in Sect. 6. The presented methods were approved by the Oregon State University IRB under protocol #IRB-2019–0067.

### 4.1 Robotic System

We used the robotic system, as more fully described in Sect. 3, for the long-term deployment.

### 4.2 Study Design

To understand participants’ usual break-taking behaviors before introducing an intervention, and to acclimate users to the system during the course of the study, we followed a two-month-long, single-case-style (ABA) design [48] similar to approaches used by leading recent human-robot interaction work [36], which allows each participant to act as their own control. This design includes pretest/baseline (A), test/intervention (B), and posttest/retention (A) phases, which are further outlined below:

- *Baseline:* two-week initial phase during which the robotic system was present but stationary.
- *Intervention:* one-month phase consisting of interactions with the SAR system, as further described in Sect. 3.
- *Retention:* two-week final phase during which the robotic system was present but stationary, like in the baseline phase.

We used this design to understand the user’s typical behavior and allow them to acclimate to the presence of the system (baseline), before introducing the robotic break-taking support (intervention), and finally checking how participant behavior may have changed after the cessation of the nudges (retention). Based on this approach, we can assess how the SAR system influences individual users, as well as how it performs across the full group.

### 4.3 Participants

The study included seven participants who spend most of their workday sitting at a desk and working with a computer. These individuals were recruited through word of mouth and snowball sampling, and were all academics associated with the university (primarily graduate students). Most participants had limited experience with robots. Four of the participants were in engineering disciplines and identified as male. The other three participants were in life sciences disciplines and identified as female. The mean age of the participants was 30 (range: 25–38 years).

### 4.4 Measures

Measurements during the deployment included information from system sensors, Likert-type self-report data, and qualitative semi-structured interviews.

We used the *system sensor logs* to collect participant behavioral data, as described below:

- *Break-taking information:* showed if and when the participant stood after each prompt. This information came from seat sensor readings.
- *Snooze inputs:* showed when the participant used the snooze button to delay a break.

Sensor logs also recorded the timing of break prompts and the specific Cozmo behaviors used for each prompt.

We used *surveys* to gauge participant opinions of and experience with robots, as well as acceptance of the system, perceptions of workload, affect, bond feelings with the robot, warmth towards the robot, robot competency, and discomfort towards the robot throughout each phase of the study. These surveys, as outlined below, used 7-point Likert scales unless otherwise noted:

- *Pre-study survey:* captured baseline robotics and break-taking technology experience, as well as perceptions related to technology acceptance. This last construct was measured using the attitude toward using technology and self-efficacy scales from the Unified Theory of Acceptance and Use of Technology (UTAUT) and the attachment, cultural context, grouping, and reciprocity scales from the Object Centered Sociality Factors, based on the work from [49]. For brevity, we later refer to this group of six items as “UTAUT” questions.
- *Weekly survey:* captured participants’ experiences after each week of the study related to workload (using questions adapted from the NASA Task Load Index [TLX] [50]), affect (using the Self-Assessment Manikin [SAM] [51] on a 9-point Likert scale), bond feelings with the robot (using the bond scale of the Working Alliance Inventory [WAI] [52]), and perceptions of robot warmth, competency, and discomfort (using the Robot Social Attributes Scale [RoSAS] [53]), in addition to queries about perceived break-taking success and work performance as modified versions of the NASA TLX performance measure. Lastly, we gathered open-ended text input about any additional thoughts or comments.
- *Closing survey:* included all questions from the weekly survey to capture to last week of the deployment, as well as all UTAUT questions from the pre-study survey, in addition to measuring the Big Five personality traits through the Ten-Item Personality Inventory (TIPI) [54] questions and general demographic questions (i.e., gender, age, hometown, ethnicity, and nationality).

To gather further context for both the system observations and survey responses, we conducted audio-recorded *semi-structured interviews* at intervals throughout the study. These conversations comprised:

- A *pre-study conversation* about participant health habits and goals.
- A *weekly check-in conversation* about current user thoughts on the system and anything notable about participant activities during the past week.
- A *closing conversation* focused on overall thoughts, suggestions, or concerns about the system.

### 4.5 Procedure

After consenting to be in the study, participants completed the pre-study survey and interview. Next, their workspace was outfitted with the system hardware, which was initially configured for the two-week baseline phase with no robot prompts. During the baseline, the participant completed weekly surveys and check-ins.

After the baseline, the system automatically transitioned to the intervention phase. The intervention lasted for four weeks, during which the robot provided break-taking prompts using the system logic described previously. The weekly surveys and check-ins continued.

Finally, the system automatically transitioned to the retention phase. During this two-week phase, the system operated in the same way as the baseline (no robot prompts), and we continued administering the weekly surveys and check-ins. The retention phase culminated with the closing survey and interview, after which the study hardware was removed from the participant’s workspace.

### 4.6 Analysis

Based on the health literature detailed previously, we knew that sitting durations of 30min or more are most detrimental to health. Accordingly, a primary test of the effectiveness of our intervention was examining how the average duration of long sitting periods changed over time for each participant. A participant with good performance using the system would in theory stand up every 30min (if not more frequently), while sitting to complete work between breaks. Accordingly, we used the seat sensor data to identify periods of extended sitting (i.e., sitting for more than 30min) and computed descriptive statistics on this data as part of our analysis. Note that breaks which lasted less than ten seconds, such as readjusting in a seat or reaching for a file folder, were not counted as a break from sitting. The number of successful prompts (i.e., the participant stood up during the robot behavior or before the next system prompt) and failed prompts (i.e., the participant did not stand during the aforementioned periods) by the system was tabulated overall and for each robot behavior category. Lastly, we calculated the mean number of prompts it took before each participant stood up to take a break.

The Likert-type results were analyzed using descriptive statistics and repeated-measures analysis of variance (rANOVA) tests across weeks with an α = 0.05 significance level. We performed Tukey’s multiple comparison tests in the case of significant main effects. These tests were run for the following survey dependent variables: attitude toward technology, self-efficacy, attachment, cultural context, grouping, and reciprocity from the UTAUT; mental demand, physical demand, temporal demand, effort, and frustration from the NASA TLX; happiness, stimulation, and control from the SAM inventory; bond perception from the WAI; warmth, competency, and discomfort from the RoSAS ratings; and our questions about perceived break-taking success and work performance.

We performed thematic analysis on the qualitative free-response comments and transcribed interview conversations. One trained coder completed a review of all the qualitative data, and a second rater coded data from 14% of the participants. Inter-rater reliability was confirmed using Cohen’s kappa. Additionally, we used the qualitative data to help track and understand any gaps or large changes in the data logged by the system.

### 4.7 Results

All seven participants remained in the study for the entire two-month duration. As is typical in academic research settings, participants spent varying amounts of time at their desks day-to-day and week-to-week. The collected data included system use information for every participant during every week except in one case; one participant missed a full week of the study due to illness during the first part of the retention phase. The quantitative behavioral and survey results appear in the following subsections.

#### 4.7.1 Objective Behaviors

**Fig. 6:**
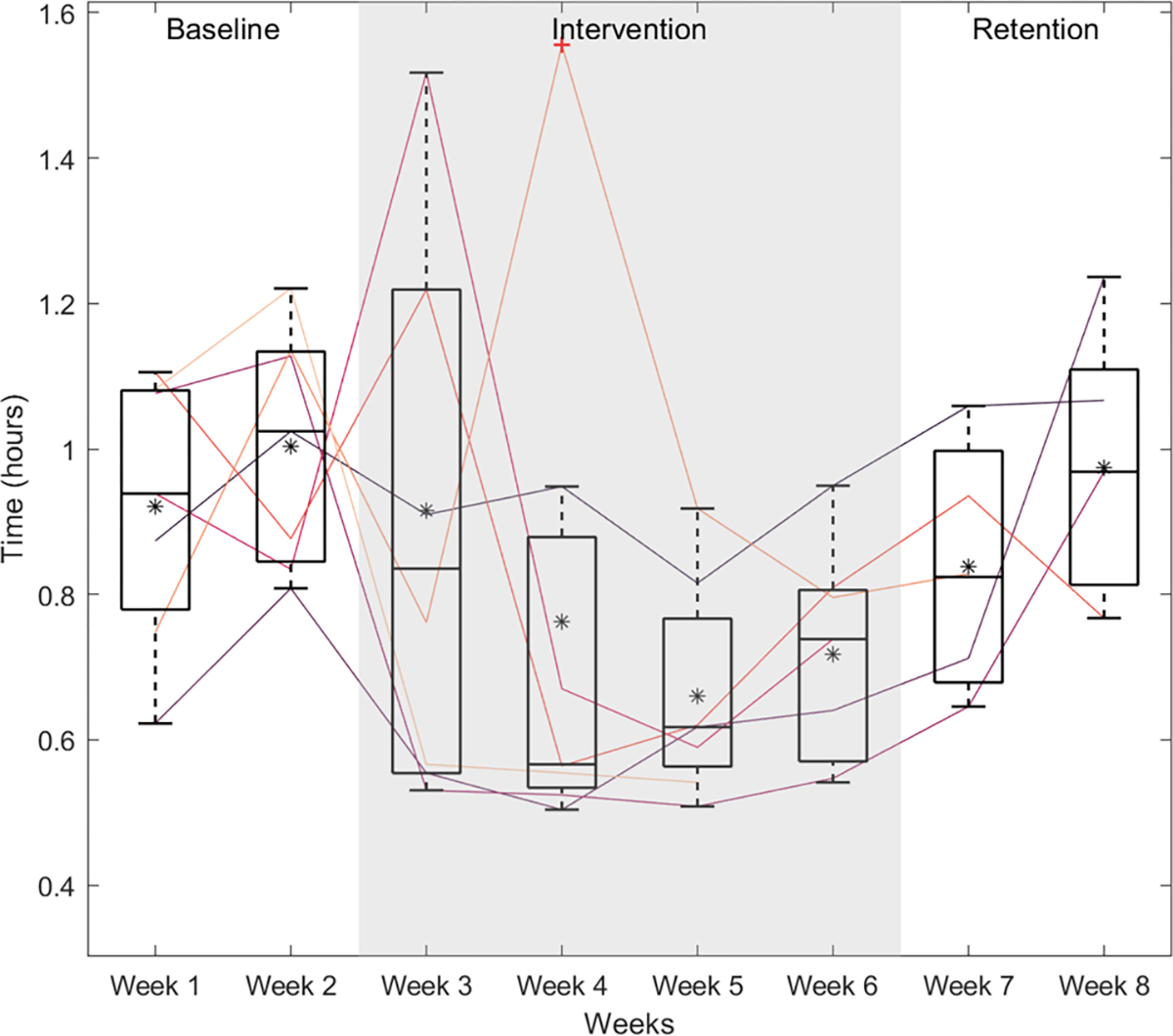
Boxplots showing the average time in hours participants spent at their desks between breaks. Each particpant’s average is plotted as a line, while boxplots show the spread across users during each week. The boxes extend from the 25th to the 75th percentiles, the middle horizontal line marks the the median, and an asterisk (*) marks the mean. The whiskers extend to the most extreme data points that are not considered outliers, and the outliers are plotted as a “+”

The average sitting duration (i.e., length of time sitting without taking a break), was 58 min (*SD* = 10) during the baseline phase, 45 min (*SD* = 17) during the intervention phase, and 55 min (*SD* = 11) during the retention phase. The average sitting duration per week for each participant are shown in Fig. 6.

Within the intervention phase, there was a large variation in total numbers of prompts. Over 60% of the time, the first prompt was successful in getting the participant to stand up, and over 90% of successes occurred within the first six prompt attempts (i.e., up to double the recommended sitting duration without a break), although there were instances of needing to prompt a participant up to seventeen times before they stood up. The median number of prompts for a given user over the course of the study was 87 (*SD* = 104), and the participant-wise total number of prompts, average number of prompts before success, and total number of incipient (i.e., first since the participant began sitting) prompts appear in Table 1.

The table information reveals a possible split between different types of users; for participants 2, 3, and 7, one nudge from the robot is typically sufficient, with very low variation for the former two participants and some variation for the latter. Other participants require more prods from Cozmo, although within this second group, there may be a split in the degree to which this is needed. Participants 4 and 5 require between two and three nudges on average, while participants 1 and 6 need a far greater number of prompts, with the greatest variability.

#### 4.7.2 Self-Reported Ratings

We describe the phase-wise averages for the survey results below, as well as the results of our statistical analysis across weeks of the study.

##### UTAUT Ratings:

The average pre-study and closing survey technology acceptance results appear in Table 2. Average ratings sat close to the center of each scale, and closing ratings tended to be similar, but slightly lower, compared to pre-study ratings. However, there were no statistically significant differences between the pre-study and closing survey responses.

##### NASA TLX Ratings:

The average baseline, intervention, and retention phase results for workload are presented in Table 3; note that only five of the six TLX questions about system use experience were administered, so each subscale is presented individually. Most responses outside of the intervention phase tended to be near the bottom of the scales. During the intervention, the responses tended to be higher for each rating. However, most of these differences were not significant; week-to-week ratings only varied significantly for mental demand (*F*(7, 42) = 3.00, *p* =.012, *η*^2^ = 0.274). Specifically, week one in the baseline phase was considered less mentally demanding than week six in the intervention phase (*M*_*di f f*_ = 1.286, *t*(9) = 4.5, *p* =.042).

##### SAM Ratings:

The average baseline, intervention, and retention phase results for user affect appear in Table 4. All of these ratings tended to be near the middle of the scale, representing moderately pleasant feelings, medium energy, and a slight leaning toward feelings of control. There were no statistically significant differences in the responses across each week.

##### WAI Ratings:

From the WAI, we administered only the questions for the bond scale, which tended to yield low ratings across each phase: baseline (*M* = 1.88, *SD* = 0.61), intervention (*M* = 2.32, *SD* = 0.76), and retention (*M* = 2.39, *SD* = 0.98), as gathered using a seven-point Likert scale. (Since we administered just one WAI scale, we omit showing this single row of data in a corresponding tabular form.) There were no statistically significant differences in the responses across each week.

##### RoSAS Ratings:

The average baseline, intervention, and retention phase results for social perception of the SAR system are presented in Table 5. Feelings about the robot’s warmth and competence tended to be above the center point of the respective scales, and discomfort with the system was generally low. There were no statistically significant differences in the responses across each week.

##### Additional Performance Ratings:

The average baseline, intervention, and retention phase results for considered types of user performance appear in Table 6. Participants overall tended to feel successful in taking breaks and performing work, though the average rating of break-taking success was below the scale midpoint for the retention phase specifically. However, there were no statistically significant differences in the responses across each week.

#### 4.7.3 Qualitative Results

The thematic analysis yielded a Cohen’s kappa inter-rater reliability of 0.78, which shows substantial agreement. A list of codes and related counts appears in Table 7.

The analysis found that prior to the study, over half of participants did not take breaks except to get food or use the restroom, but all but one of the participants mentioned the potential health or productivity benefits of taking breaks. Only two participants had workplace practices that promoted regular breaks from sitting. When discussing the value of break-taking as well as their own lack of break-taking, participants mentioned a lack of the needed “conviction” to implement break-taking, as well as previous attempts at break-taking that ebbed away over time. For example, participant 4 discussed how they “know the importance of taking breaks,” but they “just don’t practice what [they] preach.” Further, participant 1 noted a past “decision […] in the fall to try to take breaks every day” which tapered off due to “the reality of schedules.” We also heard about non-physical types of breaks, such as seated “mental” breaks for things like checking their phone. Additionally, while describing their experiences with break-taking and healthy workplace practices, participants described their posture practices using a variety of descriptors such as “good” or “bad,” or providing more nuanced descriptions, like “starting with good posture and just leaning further back with time.”

participants primarily viewed Cozmo as either toy-like or pet-like, and (contrary to perceptions discussed later on, in the follow-on data collection results), no participants viewed Cozmo as tool-like. Toy-like comparisons focused on expected interaction behaviors such as participant 4’s assertion that the robot should automatically be snoozed when lifted, since “if you hold a toy, it’ll stop.” Participant 2 lamented that they “can’t really negotiate” breaks with Cozmo. Participants 1 and 4 compared Cozmo’s responses to their cats, although one mused that “usually with cats, if I just push them away a couple of times, they get the idea and go away.”

When it came to the interactions with Cozmo, participants were concerned about it possibly disturbing other people working around them in the office. For example, participant 2 noted feeling “self-conscious about how loud Cozmo is because [their] office is supposed to be quiet.” Participants also mentioned noticing or responding to the incidental noise (such as motor noise and sound caused by physical interaction with the environment) of Cozmo before the prompt (e.g., “it’s like if you hear it come out, I’m like, okay, I need to go” [participant 7]), and although the system provided a response (in the form of a positive Cozmo expression and head nod) when participants stood up, only one of the participants (participant 6) noticed this signaling behavior, noting that “when I stand up, it looks like [Cozmo is] smiling”. Related to the topic of video-recording in the robotic system, three participants had reservations (e.g., participant 4’s quip that “my expression the first day was really bad” and they were embarrassed later after remembering that the camera was recording).

Three of the participants commented during the retention phase that they felt more awareness of their sitting time (e.g., participant 5 mused that they frequently felt that “okay, it’s really beyond the time, the period I should sit there”). Likewise, participant 3 mentioned that “every now and then […] I [felt] like the system would have turned on right now.”

### 4.8 Summary of Key Findings

The results show a tendency for our proposed intervention to hold promise; the trend was for the intervention sitting behaviors to be shorter than sitting lengths during the baseline and retention periods. It seems that the robot’s behaviors can serve as a helpful nudge. At the same time, there is clearly variation across participants, resulting in overall variability in responses to prompts. For example, a subset of participants were near-perfect system users who stood almost every time they received a prompt, while a different group was quite challenging to encourage to stand. We also did not see a positive trend in the self-report results, but these responses did tend to show a larger variation during the intervention than during the baseline phase. These variations (in both the behavioral and self-reported data) imply that a single nudging model for the robotic system may not be well aligned with all users. The thematic analysis results included glints of the system’s effectiveness at encouraging breaks and even yielding habit-like results, although some of the pet metaphors show a common desire for the system to better account for participant-specific preferences when determining how and when to supply a prompt. Based on the results of this first study, we realized that important next steps for successfully modeling break-taking interactions included the need to gather both more observations of robot nudges and data from more participants for a better understanding of typical system user archetypes.

## 5 Follow-on Data Collection

Based on the variety of user responses to the SAR system in our long-term deployment, we sought to gather a larger dataset and to understand if the same types of participant responses generalized more broadly. Accordingly, our follow-on data collection used a similar procedure, but with a shorter-term use period to allow for us to work with a larger set of participants. By using a similar procedure and set of measures to the previous study, we could also assess whether participant responses to the system were similar to reactions from the intervention phase of the long-term study in this new round of data collection. The presented deployment was approved by the Oregon State University IRB under protocol #IRB-2019–0067.

### 5.1 Robotic System

We made a single specific change to our SAR system compared to the long-term deployment: we removed the webcam. This update was made to address self-consciousness from selected participants in the long-term deployment, who described feeling “watched” or not wanting the research team to “judge them later.” We believed that the resulting behavioral data would be more authentic and show whether the participant behavior groupings persisted even without this feeling of close observation.

### 5.2 Data Collection Design

To augment our overall set of participant responses to the robotic system and gain insights about more users’ personal experiences with the system, we shortened the deployment length and de-emphasized the single-case-style design in this follow-up work. For length of data collection, we wanted to still capture interactions over a much longer period than a typical human-robot interaction study, while relaxing some of the resource-intensiveness brought about by a full two months of deployment. Accordingly, we shortened the intervention period of deployment to one week and removed the baseline and retention phases.

### 5.3 Participants

We recruited 14 participants, none of whom were participants in the initial study, for the follow-on data collection. All but two participants had advanced experience with robots, and participants primarily identified as men (11 men, 3 women). The participants had a mean age of 24 (range: 20–31 years).

### 5.4 Measures

Our measures for the week-long data collection comprised a subset of those from the long-term deployment. We used the same *system sensor log* measures as well as the same *pre-study* and *closing* surveys and conversations.

### 5.5 Procedure

The timing of the deployment for all participants was from Monday to Friday during their selected week of enrollment. After consenting, participants completed the pre-study survey and interview. Next, their workspace was outfitted with the system hardware, configured to operate in the intervention mode using the system logic described in Sect. 3. At the end of the deployment, participants would complete the closing survey and interview, after which the robotic system hardware was removed from their workspace.

### 5.6 Analysis

The analysis methods for the sitting logs were the same as those of the long-term deployment, as described in Sect. 4.6. We also performed a similar rANOVA test to the one described previously for the UTAUT results, and we used descriptive statistics to understand the other survey feedback, which were now just reported at a single time point.

We again performed thematic analysis on the qualitative free-response comments and transcribed interview conversations. One trained coder completed a review of all the qualitative data, and a second rater coded approximately 14% of participants. Inter-rater reliability was confirmed using Cohen’s kappa.

### 5.7 Results

All 14 participants remained in the data collection for the entire week-long duration. As is typical in academic research settings, participants spent varying levels of time at their desks, but all participants worked at their desks for at least one full day during the deployment duration.

#### 5.7.1 Objective Behaviors

Like in the long-term deployment, there was a large variation in the total number of times a participant was prompted. The number of prompts necessary for a participant to stand up had a long tail: over 47% of the time the first prompt was successful in getting the participant to stand up, and over 90% of successes occurred within the first six prompt attempts (i.e., up to double the recommended sitting duration without a break), although there were instances of needing to prompt a participant up to fifteen times before they stood up. The median number of prompts for a given user over the course of the data collection was 32 (*SD* = 18), and the participant-wise total number of prompts, average number of prompts before success, and total number of incipient (i.e., first since the participant began sitting) prompts appear in Table 8.

As in the long-term deployment, the table information reveals a possible split between different types of users; some participants responded quickly and reliably, and others were less responsive and more variable in their behaviors. This trend appears to generalize regardless of the overall number of prompts a participant experienced.

#### 5.7.2 Self-Reported Ratings

We describe the averages for the survey results below, as well as the results of our statistical analysis. This information helps us to compare trends in participant perceptions and experiences during the follow-on data collection to those of the long-term deployment.

##### UTAUT Ratings:

The average pre-study and closing survey technology acceptance results are presented in Table 9. Similarly to in the long-term deployment, these ratings tended to be above the midpoint of each scale, and there were no statistically significant differences between the pre-study and closing survey responses.

##### NASA TLX Ratings:

The mean ratings for each considered workload subscale were mental demand at 2.14 (*SD* = 1.35), physical demand at 1.79 (*SD* = 0.97), temporal demand at 2.79 (*SD* = 1.63), effort at 3.57 (*SD* = 1.45), and frustration at 3.00 (*SD* = 1.66), as measured on seven-point Likert scales. These evaluations tended to be in a similar scale range, but slightly lower in magnitude, compared to the analogous ratings from the long-term deployment’s intervention phase.

##### SAM Ratings:

Participants rated their mean happiness as 3.29 (*SD* = 1.53), stimulation as 3.86 (*SD* = 1.30), and control as 5.64 (*SD* = 1.80) on nine-point Likert scales. These evaluations tended to be lower than in the long-term deployment, but still represented values near the center or low center of each scale.

##### WAI Ratings:

The WAI bond questions yielded a mean rating of 2.89 (*SD* = 0.89), as measured on a seven-point Likert scale. This result is similar in magnitude to the same ratings for the intervention phase of the long-term deployment.

##### RoSAS Ratings:

The ratings of robot social attributes included a mean warmth of 4.40 (*SD* = 0.63), competence of 4.26 (*SD* = 1.04), and discomfort of 2.10 (*SD* = 0.81), as reported on seven-point Likert scales. The former two ratings tended to be higher than those seen in the long-term deployment, while the level of discomfort appeared to be very similar.

##### Additional Performance Ratings:

Participants rated their mean break-taking success as 4.64 (*SD* = 1.55) and work performance as 5.07 (*SD* = 1.07) on the related seven-point Likert scales. These values tended to be similar to the evaluations seen in the long-term deployment.

#### 5.7.3 Qualitative Results

The thematic analysis yielded a Cohen’s kappa inter-rater reliability is 0.86, which shows excellent agreement. A list of codes and related counts appears in Table 10.

The analysis found that prior to the study, half of participants did not take breaks except to get food or use the restroom, and only two of the participants mentioned the potential health or productivity benefits of taking breaks. Just two participants had workplace practices that promoted regular breaks from sitting. Interestingly, one of these two participants (participant 20) talked about how their regular break practices were “too much sometimes” because they felt that they “should be sitting at one desk and staying focused and working for like 2 to 3 [hours].” Participant 23, the other break-taker, recognized the value of their regular breaks, but noted that they sometimes heeded and sometimes ignored their current break-taking aid, “this watch which buzzes every [30min] if I’m not active.” Participants who did not take breaks included participant 22 who mentioned that “once I’m working I tend to try to keep working” and participant 17 who noted only getting up when they “need to get another glass of water.” Self-assessments of sitting posture included participant 14’s quip that they “look like a shrimp [while working]” and participant 16’s statement that they are “not a board, but pretty straight up.”

Nine participants viewed Cozmo as pet-like, with participant 24 in particular talking about “sometimes [looking] over at what the Cozmo was doing and then kind of pet-not pet[ting] but like touch[ing] it.” Counter to our observations in the long-term study, over two-thirds of participants viewed Cozmo as tool-like (e.g., Cozmo “looks more like a car [which] doesn’t seem interactive [socially]” [participant 13]), and only a third of participants viewed Cozmo as toy-like. Similar to in the long-term deployment, participants occasionally struggled with trying to communicate that “right now I really do need to stay here” (participant 19), when important periods of focus coincided with a Cozmo nudge.

When it came to the interactions with Cozmo, fewer participants were concerned about the robot possibly disturbing other people working in the office, but concerns that did arise were still primarily attributed to the incidental noise of the system (e.g., participant 21 found themselves “worried about other people in the lab hearing it move around and being annoyed by it”). Almost half of participants mentioned noticing or responding to the incidental noise of Cozmo before the prompt, with some participants noting that they “didn’t realize it was going to be that loud.” Almost half of participants noticed the programmed system response (a positive expression and head nod) when they stood up, with several of these users expressing appreciation of the movement, such as participant 19 noting that they “loved the stupid nod” and that “it was way too effective.” Participant 22 likewise noted that once they noticed this social cue, they started standing up “just to look at [Cozmo] nod.” Since the follow-up effort’s design did not include a camera for video-recording or a retention phase, the two codes related to these concepts were not part of the thematic analysis in this section.

### 5.8 Summary of Key Findings

Our week-long deployment results were similar to the results of the intervention phase from the long-term deployment. These results show large variations both in overall numbers of prompts, as well as the number of prompts necessary for participants to stand up. The variation in self-report responses is also large, and both the average and spread of responses are generally similar to those reported in the intervention phase of our long-term deployment. The qualitative results included similar themes as the long-term deployment, although the frequency of perception of the system as tool-like was much higher and toy metaphors were less common, perhaps due to the higher robotics experience of the new participant group. Notably, however, these participants often noticed Cozmo’s social nod, and some even felt compelled to adopt better workplace practices simply to elicit this behavior. Within the set of 14 additional participants, we saw hints of similar types of participant archetype groupings as in the initial long-term deployment. These groupings of participant reactions imply that a single generalized system behavior policy might be insufficient, as further investigated in Sect. 6. On the other hand, the rough groupings of participant behaviors may reveal a shortcut to model personalization based on typical user archetypes. We build on these insights to propose and assess two different approaches to model formulation in the following section.

## 6 Robot Behavior Model

We began this project with the goal of formulating behavior models that would lead to the success of robot nudges, such as our break-taking robot intervention. This aim led us to conduct a first long-term study to assess many interactions (as well as potential changes in interaction over time), in addition to a follow-on data collection to supplement the overall size and number of system users in our dataset. The results of both the initial study and the follow-up data collection showed apparent differences in the number of prompts necessary to lead users to take a break. Therefore, this section proposes a general model, considers the user types observed across the two studies, and assesses how the model would perform, as well as how it may need to be adjusted, across the groups.

### 6.1 General Model Formulation

Based on our past related work in [4], we knew that user state at the time of a robot prompt could have an important impact on the reception of a break-taking robotic system. Accordingly, to advance the success of robot nudges beyond the small improvement offered in this manuscript’s deployments (i.e., using a keyboard sensor as a minor adjustment method for break timing), we decided to use reinforcement learning to develop a more sophisticated policy for robotic break-taking nudges, building on the work of [33], which modeled user attention toward a robot via online learning during shorter interaction sessions. Our system included sensors which return clear information about the user state, and the robot’s set of behaviors spans a rich but simple-to-define action space. Accordingly, interaction scenarios with our break-taking SAR system can be discretized into a relatively small set of known state-action pairs. Thus, we formulate the behavior response problem for the system as a Markov decision process (MDP) model.

At a high level, the MDP is defined by a structure of finite world states and actions, in addition to state-transition probabilities and a cost-reward function that we use to determine an optimal behavior policy for our system. The world state of our system is defined based on the state of the user, while the actions are the robot behavior categories defined in Sect. 3. Our state-transition probabilities are determined using the two rounds of robot deployment data, which included 1,256 prompt interactions and 497 successful prompts. Each participant’s raw scores were converted to normalized probabilities before combining across participants to prevent participants with higher numbers of prompts from being dominant.

#### 6.1.1 State Space

For our MDP state space, the state *s* is defined by the tuple {*i*, *b*, *u*, *d*}, including prompt attempt number *i*, button pressed value *b*, participant standing value *u*, and standing duration *d*. We discretized each of these variables as follows:

- *i* = {1,2,3,4,5,6} - This count represented the current prompt attempt number, capped at six attempts since in the vast majority (> 90%) of instances, users stood by the sixth prompt across both studies.
- *b* = {True,False} - This value represented whether or not the participant had pressed the snooze button during the previous robot nudge.
- *u* = {True,False} - This value represents whether or not the participant is standing up. This variable helps to determine if the system scenario is in a terminal state.
- *d* = {1,2} - This value represents if the standing duration of the user is short (five minutes or less) or long (beyond five minutes), where breaks of at least five minutes are known to be beneficial [55]. This threshold was not used during the human subjects studies; it was used just here to define preferential behaviors within the reward state. Like the past variable, it is relevant in the terminal state; *d* helps to later define the reward formulation.

The initial system statealways begins with*i* = 1, *b* = *False* (since there has not yet been an opportunity to press the snooze button), and *u* = *False*.

#### 6.1.2 Actions

Actions, denoted as *a*, are defined by the previously described robot behavior categories. As a reminder, these eight possible action (i.e., robot behavior) options are Active (A), Pleasant Active (PA), Pleasant (P), Pleasant Inactive (PI), Inactive (I), Unpleasant Inactive (UI), Unpleasant (U), Unpleasant Active (UA). Each action has its own associated cost value, as further described below.

#### 6.1.3 Cost-Reward Function

Our cost-reward function is the additive combination of distinct cost and reward functions, as detailed in this subsection.

##### Cost Function:

Based on past work that shows the sound level of assistive workplace robotic systems to be potentially detrimental [8, 27] and the action footprint of the robot to come at some expense to the user experience [4, 8], the proposed cost function is determined with consideration of the invasiveness of the robot action (via incidental sound such as motor noise and sound caused by physical interaction with the environment and area of movement), as well as the prompt attempt count *i*.

To capture the cost associated with incidental sound in each robot action, we used the International Organization for Standardization’s (ISO) standard 532 [56], which describes how to calculate loudness level (in sones) based on recorded sound. The audio from incidental sound for each behavior category was recorded at a sampling frequency of 16 kHz. Each recording was analyzed with MATLAB’s in-built acoustic loudness function (<monospace>acousticLoudness()</monospace>), which complies with ISO 532. The resulting cost of each robot action’s sound appears in Table 11.

To capture the cost of the movement for each robot action, we assessed the amount of desk space traversed during each prompt type. The trace of each robot action was recorded using a marker attached to the robot to determine both area traversed and cumulative distance traveled within that area. We grouped the actions into categories nominally defined by traversal area, with the exception of the Active action; while the area of the Active motion was smaller, the cumulative distance traversed within that area was double that of the other actions with similar areas. These categories were assigned cost values from zero (no motion) to three (largest area) and are presented in Table 11. Note that the relative cost of sound (compared to motion) is higher based on its fairly uniform description as a negative feature across related literature. While footprint of motion also appears to play an important role, it is a bigger factor in the case of small desk areas.

In the cost function equation (Eqn. 1), these robot action costs and the attempt number *i* play a role in determining overall cost. With respect to the prompt number, the cost is formulated to encourage variation in first prompt action, impose more costs for more invasive robot actions in middle prompts, and encourage more invasive (and ideally influential) actions for later prompts. This idea is captured in the overall cost function C(i,a) below, which includes sound cost $C_{s}(a)$, motion cost $C_{m}(a)$, and prompt attempt i.

(1) $$C(i,a)=\frac{C_{s}(a)\;+\;C_{m}(a)}{2}e^{-(i-1)/5}$$

##### Reward Function:

For maximal health benefits to the system user, it is best for users to stand as soon after the first robot nudge as possible and spend time standing or being active before returning to being seated. Accordingly, the reward function R(i,d), as shown in Eqn. 2, is determined with consideration of break length d and prompt count i. Differing lengths of break determine the base reward, r(d). While the primary goal is to encourage any break, based on related literature such as [55], we want the model to encourage behaviors that lead to longer breaks over shorter breaks. Therefore, short breaks return a reward value of seven, while long breaks lead to a reward value of 10. The reward decreases with prompt number, where the sixth prompt no longer provides any reward.

(2) R(i,d)=(6-i)*r(d)

#### 6.1.4 Results

We used Q-learning to generate a set of maximized policy actions and associated success probabilities for each system state, with transition probabilities based on the aggregate information of our user response data from both system deployments, after normalizing to provide equal weightings. This general policy appears in Table 12.

The success of the chosen maximized action decreases for the fifth prompt attempt and fails to succeed for the sixth prompt attempt. Based on average number of prompts required for the different participants for each study, and the notable drop off in success, these results led us to further explore how these participant-wise (or at least participant group-wise) trends interacted with our model.

### 6.2 Participant-Specific Modeling

As alluded to in the results for each deployment, one unexpected observation as we collected data was that participants seemed to belong to one of roughly three archetypes in their responses to the robotic system. We further clarify these user types and consider the effects of specific tactics that work best for each group in this subsection.

#### 6.2.1 Participant Groupings

To better articulate the types of system user observed during the system deployments, we sorted the participants into three groups, which we refer to as *archetypes*, based on the average number of prompts required for them to stand up and take a break from working. The resulting archetype groupings appear in Table 13 and are further described below:

- *Compliant* participants averaged between one and two prompts before taking a break. This group was often very responsive and typically stood up in response to the very first prompt.
- *Moderate* participants averaged between two and four prompts. Participants in this group sometimes responded to robot prompts right away, but sometimes required a larger amount of nudging before taking a break.
- *Resistant* participants averaged four or more prompts before standing up. These users would frequently ignore the system, and sometimes ignored up to seventeen prompts in a row.

#### 6.2.2 Archetype-Specific Results

Although we would expect adaptive system performance that is personalized to individual users over time to yield the best intervention results, accruing enough data to individualize models takes time. The proposed user archetypes provide an alternative whereby very early model customization might be possible, before collecting almost any data from a new system user. To assess how much impact this type of preliminary personalization might have compared to a general MDP model, we used Q-learning to identify the maximized policy actions for each system state using policies trained based on data from each archetype group. These new result appear next to the general model results in Table 14.

**Fig. 7:**
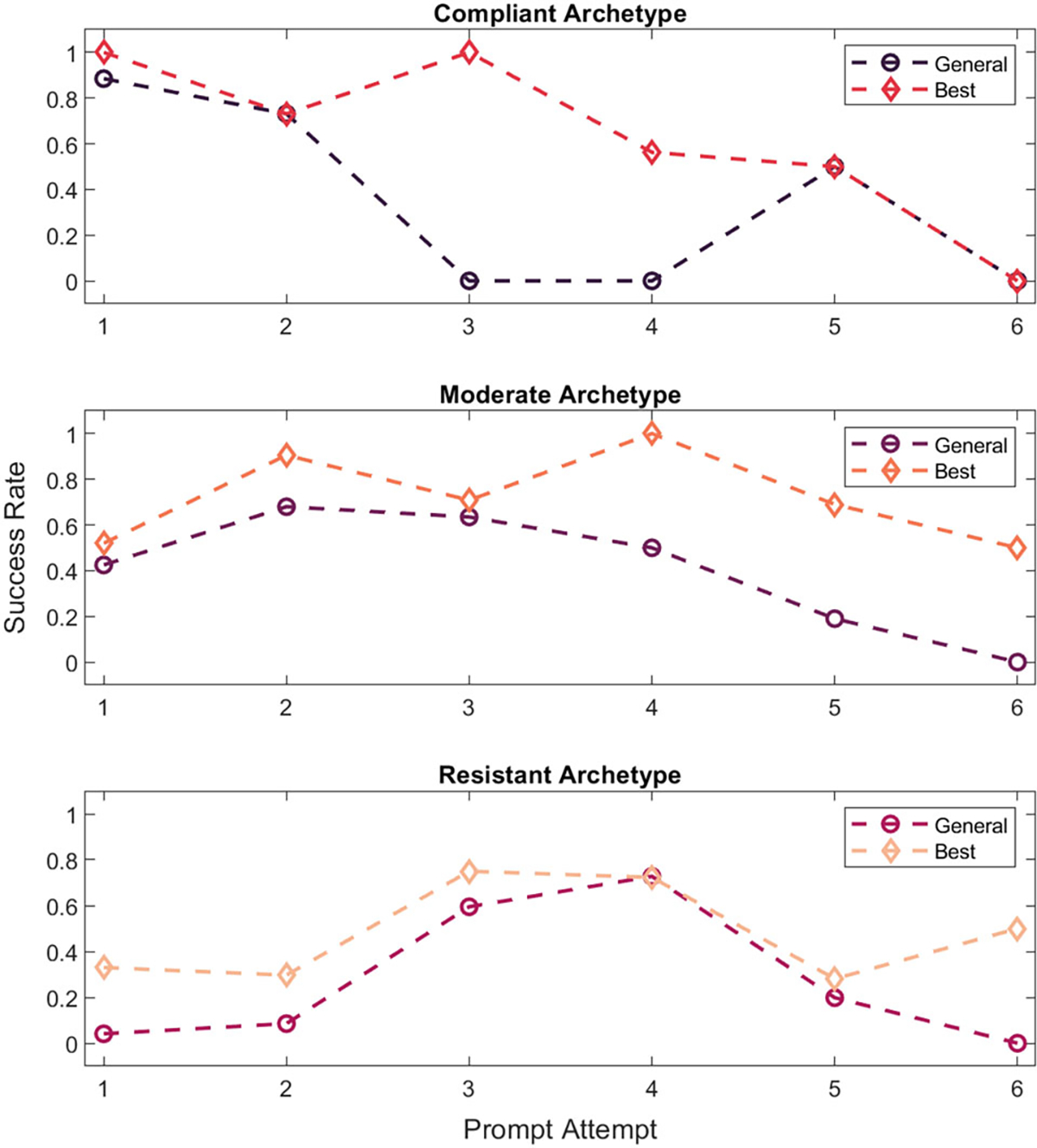
Plots comparing the success probabilities of the general maximized policy to each archetype best policy for each archetype

The recommended system behaviors for each archetype vary widely, and only match the general policy for a small number of states. This led us to wonder about the impact of archetype-specific models (compared to the general model) on system effectiveness in prompting breaks. Figure 7 displays the success probability (averaged across button state, for easy viewing) for each archetype when using the general policy, compared to each archetype-specific policy. The general policy success is well aligned for the first two prompt attempts, before beginning to deviate from the compliant and subsequently the other two archetypes. However, even when well-aligned the general policy does not show success above that of the archetypal policy.

### 6.3 Summary of Key Results

The maximized general policy may offer promise for improved user responses compared to random robot actions, but evidence of this potential was not strong. For example, the probability of success of a first system prompt was similar for the general policy compared to the average success of the first prompt across the two robot deployments. This finding, combined with the apparent user groupings hinted at by the results of each deployment, suggested that more personalization was needed for maximal SAR intervention success. Once implemented, these archetype-specific models (for compliant, moderate, and resistant system users) demonstrated benefits for the intervention; the maximized behaviors between archetypes are distinct, with only a small overlap across archetypes and compared to the general policy. Note that the lower success for the compliant archetype beyond the second prompt is strongly influenced by the available data; overall, there were only nine instances of members of this group remaining seated after the second prompt. In contrast, both the resistant and moderate archetypes have a broader distribution of prompt counts; thus, the later results for these archetypes are likely apt.

## 7 Discussion

The SAR intervention tended to lead to shorter continuous sitting periods in the initial long-term deployment, and results also showed apparent groupings in the way users responded to the robot prompts. The follow-on data collection results reflected these same groupings. We used the results of these two deployments to generate a general MDP policy, as well as more archetypal policies designed to fit the needs of the three identified participant groups. We found that while a general policy may be useful as a naïve starting point, archetype specific strategies are a better choice across all participant groups within our model analysis. For the moderate and resistant archetypes, this more personalized approach seems to offer a particularly big performance boost.

Although qualitative data hinted at the need for personalization to individuals’ styles, and even moods and workflows, there was a conflicting trend wherein users were also wary of invasive data collection techniques (e.g., video recording). Strategies such as user archetype modeling and less-invasive sensing methods such as the occupancy sensor used in this work hold promise for helping to navigate this challenging tension. More follow-up work is needed to determine if robotic systems that nudge can encourage habit formation, but anecdotes from the long-term deployment suggest that after-effects from our intervention led to more awareness of uninterrupted periods of sedentary behavior.

### 7.1 Design Implications

The alignment of types of participant responses across both the long-term and follow-on system deployments has promising implications about patterns of user needs in SAR-mediated break-taking support (and possibly beyond). While a general policy may serve as a reasonable default, the ability to categorize participants into distinct archetypes using a single, minimally invasive sensor and personalize the robot’s policy accordingly provides a promising avenue for improving SAR system success while accounting for user concerns related to data collection. Our methods could be used for offline training and even model personalization after a brief trial interaction with the robotic system.

Further, our MDP model generation process included concrete proposed measures of impact for non-verbal embodied system behaviors. Methods for measuring robot sound levels and amounts of movement can support a more structured process for quantifying robot affect and potentially applying and transferring our proposed model across systems with different SAR agents. Beyond the robotic break-taking support space, the introduced technique for quantifying aspects of non-verbal robot behavior might inform new methods for proposing and automatically validating robot affect in expressive robotic systems generally.

### 7.2 Key Strengths and Limitations

Strengths of this work include the length of studied robot interventions, the relatively noninvasive sensing used by the SAR system, and the modeling tactics further highlighted in the previous subsection. In human-robot interaction research, deployments extending beyond an hour or so are unusual, and experiments lasting a month or more are especially rare. At the same time, efforts like ours which surpass brief-interaction-based studies are important for understanding potential effects of novelty and for collecting sufficient data to advance the state of modeling in human-robot interaction. Past participants both within and beyond our research efforts have expressed hesitance about having a camera in their day-to-day spaces; thus, our design of interaction models that do not rely on cameras (or access to a user’s personal or corporate electronic devices, for that matter) can support broader relevance and adoption of everyday SAR systems for healthy nudging. Lastly, as already highlighted in Sect. 7.1, the participant archetype-specific models and non-verbal behavior quantification proposed in this work can benefit the field both within and beyond the robot nudging space.

At the same time, this work was not without limitations. For example, without a larger SAR system fleet and research team, we were limited in the number of participants we could recruit. We aimed for a sample that was sufficient for informing the proposed models, but more data collection would be needed to reach conclusive empirical findings about the nudging intervention and its benefits. Further, the participant group was mostly male and tended to include individuals with moderate-to-high levels of technology experience. Recruiting a sample more representative of general consumer electronic device users would help to ensure that the observed findings can generalize as desired. Lastly, our proposed policies have only been tested post hoc within the presented work. Future real-world application and testing of the policies will be essential for fully understanding their potential impact on SAR system success.

### 7.3 Conclusions

While we began this work based on an interest in exploring break-taking SARs and understanding the impact of such systems over longer-term deployments, we became curious along the way about how participant needs might be modeled, both generally and within more personalized archetypes. This emergent interest was fueled by the user archetypes that presented themselves during both the long-term and short-term system deployments. After observing that our SAR intervention tended to be effective, but appeared to work better for some participants than others, we used the collected user response data and participant archetype groupings to model both general and group-specific system policies. In initial testing, we found that archetype-specific policies performed better than the general policy for each user group, with especially noticeable benefits for the moderate and resistant groupings. Future work is needed to understand the influence of the proposed policies in real-world deployments, but overall, we believe that this work can help to advance the state of decision-making in nudge-related SAR research, in addition to assistive and expressive robotics more broadly.

## Figures and Tables

**Table 1:** Intervention phase data for total number of prompts experienced by each participant, *M*(*SD*) number of prompts needed for the participant to stand up, and total number of incipient prompts

Participant	Total prompts	Prompts to stand	Incipient prompts

01	343	4.1 (2.9)	79
02	20	1.2 (0.4)	15
03	78	1.0 (0.2)	75
04	79	2.6 (2.0)	28
05	87	2.1 (2.1)	41
06	113	5.0 (5.3)	20
07	87	1.3 (1.1)	63

**Table 2:** Results for each UTAUT measure for the pre-study and closing surveys for the long-term study, reported as *M*(*SD*)

Measure	Pre-study	Closing

Attitude to technology	4.93 (1.31)	4.36 (1.25)
Self-efficacy	4.77 (1.58)	4.29 (1.95)
Attachment	4.25 (1.53)	3.64 (1.95)
Cultural context	3.71 (1.25)	3.43 (1.27)
Grouping	3.69 (1.16)	3.29 (1.48)
Reciprocity	3.54 (1.52)	3.00 (1.80)

All values were on a seven-point Likert scale

**Table 3:** Results for each TLX measure for each phase for the long-term study, reported as *M*(*SD*)

Measure	Baseline	Intervention	Retention

Mental demand	1.43 (0.65)	2.61 (1.31)	1.43 (0.94)
Physical demand	1.29 (0.61)	2.00 (1.12)	1.36 (0.93)
Temporal demand	1.21 (0.58)	3.50 (1.93)	2.07 (1.73)
Effort	2.21 (1.48)	4.14 (1.21)	3.57 (1.91)
Frustration	1.21 (0.58)	3.54 (2.10)	2.14 (1.79)

All values were on a seven-point Likert scale

**Table 4:** Results for each SAM measure for each phase for the long-term study, reported as *M*(*SD*)

Measure	Baseline	Intervention	Retention

Happiness	4.00 (0.88)	4.71 (1.96)	4.29 (1.38)
Stimulation	5.64 (1.15)	4.50 (1.40)	5.43 (2.31)
Control	6.36 (1.39)	5.36 (2.25)	6.14 (2.07)

All values were on a nine-point Likert scale

**Table 5:** Results for each RoSAS measure for each phase for the long-term study, reported as *M*(*SD*)

Measure	Baseline	Intervention	Retention

Warmth	4.35 (0.46)	3.76 (1.56)	3.85 (1.40)
Competence	4.33 (0.53)	3.66 (1.45)	4.06 (1.64)
Discomfort	1.70 (0.40)	2.20 (1.16)	1.76 (0.89)

All values were on a seven-point Likert scale

**Table 6:** Mean results for our additional ratings for each phase for the long-term study, reported as *M*(*SD*)

Measure	Baseline	Intervention	Retention

Break-taking success	4.00 (0.68)	4.32 (1.49)	3.36 (1.39)
Work performance	5.43 (0.94)	5.21 (0.99)	5.43 (0.94)

All values were on a seven-point Likert scale

**Table 7:** Thematic analysis codes and counts of participants who mentioned each code (out of the seven total participants)

Code	Participant count

Mentioned benefit of breaks	6
Said Cozmo is toy-like	5
Did not previously take breaks	4
Said Cozmo is pet-like	4
Worried about disturbing others	4
Noticed noise before prompt	4
Claimed good posture	3
Claimed hunching posture over time	3
Had awareness of sit time post	3
Worried about video recording	3
Stretching/walking breaks	2
Previously took routine standing breaks	2
Previously took “mental” seated breaks	1
Claimed “bad” posture	1
Noticed Cozmo’s nod reaction	1
Said Cozmo is tool-like	0

**Table 8:** Intervention data for total number of prompts experienced by each participant, *M*(*SD*) number of prompts needed for the participant to stand up, and total number of incipient prompts

Participant	Total prompts	Prompts to stand	Incipient prompts

11	6	1.0 (0.0)	6
12	24	1.8 (1.1)	13
13	36	7.2 (7.3)	5
14	8	4.0 (2.0)	2
15	48	1.6 (1.2)	30
16	50	2.5 (2.3)	20
17	28	3.5 (3.8)	8
18	20	1.1 (0.4)	15
19	47	2.3 (3.3)	20
20	72	4.7 (5.0)	14
21	31	2.2 (1.7)	14
22	29	2.4 (3.0)	12
23	49	4.6 (3.7)	10
24	32	4.0 (4.7)	8

**Table 9:** Results for each UTAUT measure for the pre-study and closing surveys for the follow-on data collection, reported as *M*(*SD*)

Measure	Pre-study	Closing

Attitude to technology	4.68 (1.10)	4.29 (1.58)
Self-efficacy	5.50 (0.78)	5.62 (1.22)
Attachment	4.25 (1.01)	4.36 (1.47)
Cultural context	3.88 (1.06)	3.93 (1.10)
Grouping	3.62 (0.69)	3.79 (0.94)
Reciprocity	3.71 (1.14)	3.71 (1.31)

All values were on a seven-point Likert scale

**Table 10:** Thematic analysis codes and counts of participants who mentioned each code (out of the fourteen total participants)

Code	Participant count

Said Cozmo is tool-like	10
Said Cozmo is pet-like	9
Did notpreviously take breaks	7
Noticed noise before prompt	6
Noticed Cozmo’s nod reaction	6
Previously took stretching/walking breaks	5
Worried about disturbing others	5
Said Cozmo is toy-like	5
Claimed “bad” posture	4
Claimed hunching posture over time	3
Claimed good posture	3
Previously took “mental” seated breaks	3
Mentioned benefit of breaks	2
Previously took routine standing breaks	2

**Table 11:** Each action’s calculated sound cost and defined movement cost

a	C_s_	C_m_

A	18.3	2
PA	19.6	3
P	15.7	1
PI	11.7	0
I	7.1	0
UI	13.8	1
U	16.0	1
UA	22.5	2

**Table 12:** The general maximized policy and the probability of success for the maximized policy action for each state, aggregated across all participants

Attempt	Button	Action	Success probability

1	False	UI	0.518
2	True	PA	0.405
2	False	UA	0.437
3	True	U	0.611
3	False	U	0.312
4	True	PA	1.0
4	False	A	0.469
5	True	PI	0.206
5	False	A	0.259
6	True	PA	0.0
6	False	P	0.0

**Table 13:** The participants for each archetype and their associated average number of prompts

Compliant		Moderate		Resistant	
		
P	Average	P	Average	P	Average

02	1.2	04	2.6	01	4.1
03	1.0	05	2.1	06	5.0
07	1.3	16	2.5	13	7.2
11	1.0	17	3.5	14	4.0
12	1.8	19	2.3	20	4.7
15	1.6	21	2.2	23	4.6
18	1.1	22	2.4	24	4.0

**Table 14:** The maximized actions for the general policy, as well as for the archetype-specific policies. Note that the General policy row reflects the same information as the policies selected in Table 14, but flipped from a column to a row orientation

Attempt	1	2		3		4		5		6	
					
Button	False	True	False	True	False	True	False	True	False	True	False

General	UI	PA	UA	U	U	PA	A	PI	A	PA	P
Compliant	P	PA	UA	PA	A	A	UA	A	A	A	A
Moderate	I	P	UA	UA	PA	I	A	I	A	PA	A
Resistant	A	I	P	U	UI	PA	U	U	I	U	P

## Data Availability

The datasets generated during the current study are not available for sharing based on the current IRB approval. If required, the research team can request a revision to the protocol to be able to share these materials.

## References

[1] Owen N, Healy GN, Matthews CE, Dunstan DW (2010) Too much sitting: the population health science of sedentary behavior. Exerc Sport Sci Rev 38(3):105–113 20577058 10.1097/JES.0b013e3181e373a2 PMC3404815

[2] Morris AS, Mackintosh KA, Owen N, Dempsey PC, Dunstan DW, McNarry MA (2021) Rise and recharge: exploring employee perceptions of and contextual factors influencing an individual-level e-health smartphone intervention to reduce office workers’ sedentary time at work. Int J Environ Res Public Health 18(18):9627 34574551 10.3390/ijerph18189627 PMC8467510

[3] Mark G, Iqbal ST, Czerwinski M, Johns P (2014) Bored Mondays and focused afternoons: the rhythm of attention and online activity in the workplace. In: Proceedings of the CHI conference on human factorsincomputingsystems.CHI’14,pp3025–3034.Association for Computing Machinery, New York, NY, USA

[4] Zhang BJ, Quick R, Helmi A, Fitter NT (2020) Socially assistive robots at work: making break-taking interventions more pleasant, enjoyable, and engaging. In: IEEE/RSJ international conference on intelligent robots and systems (IROS), pp 11292–11299

[5] Bainbridge WA, Hart JW, Kim ES, Scassellati B (2011) The benefits of interactions with physically present robots over video-displayed agents. Int J Soc Robot 3(1):41–52

[6] Kidd CD, Breazeal C (2008) Robots at home: understanding long-term human-robot interaction. In: IEEE/RSJ international conference on intelligent robots and systems (IROS), pp 3230–3235

[7] Jafarinaimi N, Forlizzi J, Hurst A, Zimmerman J (2005) Break-away: an ambient display designed to change human behavior. In: Extended abstracts of the CHI conference on human factors in computing systems. CHI EA ’05, pp 1945–1948

[8] Sabanovic S, Reeder S, Kechavarzi B (2014) Designing robots in the wild: in situ prototype evaluation for a break management robot. J Hum Robot Interact 3(1):70

[9] Kaur H, Williams AC, McDuff D, Czerwinski M, Teevan J, Iqbal ST (2020) Optimizing for happiness and productivity: Modeling opportune moments for transitions and breaks at work. In: Proceedings of the CHI conference on human factors in computing systems. CHI ’20, pp 1–15. Association for Computing Machinery, New York, NY, USA

[10] Fukushima N, Machida M, Kikuchi H, Amagasa S, Hayashi T, Odagiri Y, Takamiya T, Inoue S (2021) Associations of working from home with occupational physical activity and sedentary behavior under the COVID-19 pandemic. J Occup Health 63(1):12212 10.1002/1348-9585.12212 PMC7938758 33683779

[11] Falk GE, Mailey EL, Okut H, Rosenkranz SK, Rosenkranz RR, Montney JL, Ablah E (2022) Effects of sedentary behavior interventions on mental well-being and work performance while workingfromhomeduringthecovid-19pandemic:Apilotrandomized controlled trial. Int J Environ Res Public Health 19(11):6401 35681986 10.3390/ijerph19116401 PMC9180109

[12] Fazzi C, Saunders DH, Linton K, Norman JE, Reynolds RM (2017) Sedentary behaviours during pregnancy: a systematic review. Int J Behav Nutr Phys Act 14(1):1–13 28298219 10.1186/s12966-017-0485-z PMC5353895

[13] Diaz KM, Howard VJ, Hutto B, Colabianchi N, Vena JE, Safford MM, Blair SN, Hooker SP (2017) Patterns of sedentary behavior and mortality in US middle-aged and older adults. Ann Internal Med 167(7):465–475 28892811 10.7326/M17-0212 PMC5961729

[14] Bergouignan A, Legget KT, De Jong N, Kealey E, Nikolovski J, Groppel JL, Jordan C, O’ day R, Hill JO, Bessesen DH (2016)Effect of frequent interruptions of prolonged sitting on self-perceived levels of energy, mood, food cravings and cognitive function. Int J Behav Nutr Phys Act 13(1):1–12 27809874 10.1186/s12966-016-0437-z PMC5094084

[15] Stockwell S, Schofield P, Fisher A, Firth J, Jackson SE, Stubbs B, Smith L (2019) Digital behavior change interventions to promote physical activity and/or reduce sedentary behavior in older adults: A systematic review and meta-analysis. Exp Gerontol 120:68–87 30836130 10.1016/j.exger.2019.02.020

[16] Muellmann S, Forberger S, Möllers T, Bröring E, Zeeb H, Pischke CR (2018) Effectiveness of ehealth interventions for the promotion of physical activity in older adults: a systematic review. Prev Med 108:93–110 29289643 10.1016/j.ypmed.2017.12.026

[17] Yerrakalva D, Yerrakalva D, Hajna S, Griffin S (2019) Effects of mobile health app interventions on sedentary time, physical activity,andfitnessinolderadults:systematicreviewandmeta-analysis. J Med Internet Res 21(11):14343 10.2196/14343 PMC6908977 31778121

[18] Luo Y, Lee B, Wohn DY, Rebar AL, Conroy DE, Choe EK (2018) Time for break: understanding information workers’ sedentary behavior through a break prompting system. In: Proceedings of the CHI conference on human factors in computing systems. CHI ’18, pp 1–14. Association for Computing Machinery, New York, NY, USA

[19] Shrestha N, Kukkonen–Harjula K, Verbeek J, Ijaz S, Hermans V, Pedisic Z (2018) Workplace interventions for reducing sitting at work. Cochrane Datab Syst Rev (12) 10.1002/14651858.CD010912.pub5 PMC6517221 30556590

[20] Edwardson CL, Yates T, Biddle SJH, Davies MJ, Dunstan DW, Esliger DW, Gray LJ, Jackson B, O’Connell SE, Waheed G, Munir F (2018) Effectiveness of the stand more AT (SMArT) work intervention: cluster randomised controlled trial. BMJ 363 10.1136/bmj.k3870 PMC6174726 30305278

[21] Healy GN, Eakin EG, LaMontagne AD, Owen N, Winkler EAH, Wiesner G, Gunning L, Neuhaus M, Lawler S, Fjeldsoe BS, Dunstan DW (2013) Reducing sitting time in office workers: Short-term efficacy of a multicomponent intervention. Prev Med 57(1):43–48 23597658 10.1016/j.ypmed.2013.04.004

[22] Thaler RH, Sunstein CR (2008) Nudge: improving decisions about health, wealth, and happiness. Yale University Press, New Haven

[23] Vlaev I, King D, Dolan P, Darzi A (2016) The theory and practice of “nudging”: Changing health behaviors. Public Adm Rev 76(4):550–561

[24] Klusmann V, Gow AJ, Robert P, Oettingen G (2021) Using theories of behavior change to develop interventions for healthy aging. J Gerontol Ser B 76(S2):191–205 10.1093/geronb/gbab111 34515775

[25] Conner M, Rhodes RE, Morris B, McEachan R, Lawton R (2011) Changing exercise through targeting affective or cognitive attitudes. Psychol health 26(2):133–149 21318926 10.1080/08870446.2011.531570

[26] Cesareo M, Tagliabue M, Oppo A, Moderato P (2021) The ubiquity of social reinforcement: a nudging exploratory study to reduce the overuse of smartphones in social contexts. Cogent Psychol 8(1):1880304

[27] Palinko O, Ogawa K, Yoshikawa Y, Ishiguro H (2018) How should a robot interrupt a conversation between multiple humans. International conference on social robotics. Springer, Berlin, pp 149–159

[28] Wang Y, Reiterer H (2019) The point-of-choice prompt or the always-on progress bar? a pilot study of reminders for prolonged sedentary behavior change. In: Extended abstracts of the CHI conference on human factors in computing systems. CHI EA ’19. Association for Computing Machinery, New York, NY, USA, pp 1–6

[29] Saulnier P, Sharlin E, Greenberg S (2011) Exploring minimal nonverbal interruption in HRI. In: Proceedings of the IEEE international symposium on robot and human interactive communication (RO-MAN), pp. 79–86

[30] Henning RA, Jacques P, Kissel GV, Sullivan AB, Alteras-Webb SM (1997) Frequent short rest breaks from computer work: effects on productivity and well-being at two field sites. Ergonomics 40(1):78–91 8995049 10.1080/001401397188396

[31] Dababneh AJ, Swanson N, Shell RL (2001) Impact of added rest breaks on the productivity and well being of workers. Ergonomics 44(2):164–174 11209875 10.1080/00140130121538

[32] Perlow LA (1999) The time famine: toward a sociology of worktime. Adm Sci Q 44(1):57–81

[33] Chiang Y-S, Chu T-S, Lim CD, Wu T-Y, Tseng S-H, Fu L-C (2014) Personalizing robot behavior for interruption in social human-robot interaction. In: IEEE international workshop on advanced robotics and its social impacts, pp 44–49

[34] Puranik H, Koopman J, Vough HC (2020) Pardon the interruption: an integrative review and future research agenda for research on work interruptions. J Manag 46(6):806–842

[35] Feil-Seifer D, Mataric MJ (2005) Defining socially assistive robotics. In: Proceedings of the IEEE international conference on rehabilitation robotics (ICORR), pp 465–468

[36] Scassellati B, Boccanfuso L, Huang C-M, Mademtzi M, Qin M, Salomons N, Ventola P, Shic F (2018) Improving social skills in children with asd using a long-term, in-home social robot. Sci Robot 3(21) 10.1126/scirobotics.aat7544 PMC10957097 33141724

[37] Dagan E, Fey J, Kikkeri S, Hoang C, Hsiao R, Isbister K (2020) Flippo the robo-shoe-fly: a foot dwelling social wearable companion. In: Extended abstracts of the CHI conference on human factors in computing systems. CHI EA ’20. Association for Computing Machinery, New York, NY, USA, pp 1–10

[38] Chen TL, Bhattacharjee T, Beer JM, Ting LH, Hackney ME, Rogers WA, Kemp CC (2017) Older adults’ acceptance of a robot for partner dance-based exercise. PLoS ONE 12(10):1–29 10.1371/journal.pone.0182736 PMC5646767 29045408

[39] Gouko M, Kim CH (2016) Can object-exclusion behavior of robot encourage human to tidy up tabletop? In: IEEE international conference on robotics and biomimetics (ROBIO), pp 1838–1844

[40] Caic M, Avelino J, Mahr D, Odekerken-Schröder G, Bernardino A (2019) Robotic versus human coaches for active aging: an automated social presence perspective. Int J Soc Robot 12:867–882

[41] Jelínek M, Fischer K (2021) The role of emotional expression in behavior change coaching by a social robot. In: Ali R, Lugrin B, Charles F (eds) Persuasive technology. Springer, Cham, pp 193–199

[42] Chan L, Zhang BJ, Fitter NT (2021) Designing and validating expressive cozmo behaviors for accurately conveying emotions. In: Proceedings of the IEEE international symposium on robot and human interactive communication (RO-MAN), pp 1037–1044

[43] Russell JA (1980) A circumplex model of affect. J Pers Soc Psychol 39(6):1161

[44] Tenchov K (2020) PyCozmo, 0.8.0, GitHub

[45] Rea DJ, Schneider S, Kanda T (2021)“Is this all you can do? harder!”:The effects of (im)polite robot encouragement on exercise effort. In: Proceedings of the ACM/IEEE international conference on human-robot interaction. HRI ’21, pp 225–233. Association for Computing Machinery, New York, NY, USA

[46] Preston RC, Dinsdale K, Shippy MR, Fitter NT appendix to robot-mediated nudges for workplace health: not a one-size-fits-all modeling problem. https://github.com/shareresearchteam/Robot-Mediated-Nudging 10.1007/s12369-023-01086-x PMC11377023 39239458

[47] de Graaf MMA, Ben Allouch S, van Dijk JAGM (2016) Long-term evaluation of a social robot in real homes. Interact Stud 17(3):462–491

[48] Byiers BJ, Reichle J, Symons FJ (2012) Single-subject experimental design for evidence-based practice. Am J Speech Lang Pathol 21(4):397–414 23071200 10.1044/1058-0360(2012/11-0036) PMC3992321

[49] Weiss A, Bernhaupt R, Tscheligi M, Wollherr D, Kuhnlenz K, Buss M (2008) A methodological variation for acceptance evaluation of human-robot interaction in public places. In: Proceedings of the IEEE international symposium on robot and human interactive communication (RO-MAN), pp 713–718

[50] Hart SG, Staveland LE (1988) Development of NASA-TLX (Task Load Index): Results of Empirical and Theoretical Research. In: Human mental workload: advances in psychology, vol 52, pp 139–183

[51] Bradley MM, Lang PJ (1994) Measuring emotion: the self-assessment manikin and the semantic differential. J Behav Ther Exp Psychiatry 25(1):49–59 7962581 10.1016/0005-7916(94)90063-9

[52] Horvath AO, Greenberg LS (1989) Development and validation of the working alliance inventory. J Couns Psychol 36(2):223–233

[53] Carpinella CM, Wyman AB, Perez MA, Stroessner SJ (2017) The robotic social attributes scale (RoSAS) development and validation. In: Proceedings of the ACM/IEEE international conference on human-robot interaction, pp 254–262

[54] Gosling SD, Rentfrow PJ, Swann WB (2003) A very brief measure of the big-five personality domains. J Res Pers 37(6):504–528

[55] Paulus M, Kunkel J, Schmidt SCE, Bachert P, Wäsche H, Neumann R, Woll A (2021) Standing breaks in lectures improve university students’ self-perceived physical, mental, and cognitive condition. Int J Environ Res Public Health 18(8):4204 33921094 10.3390/ijerph18084204 PMC8071424

[56] ISO Central Secretary: Acoustics - Methods for calculating loudness - Part 1: Zwicker method. Standard ISO 532–1:2017, International Organization for Standardization, Geneva, CH (2017). https://www.iso.org/standard/63077.html

